# Subcellular mapping of living cells via synchrotron microFTIR and ZnS hemispheres

**DOI:** 10.1007/s00216-018-1245-x

**Published:** 2018-07-21

**Authors:** K. L. Andrew Chan, Pedro L. V. Fale, Ali Atharawi, Katia Wehbe, Gianfelice Cinque

**Affiliations:** 1 Institute of Pharmaceutical Science, School of Cancer and Pharmaceutical Science, King’s College London, London, SE1 9NH UK; 2 Center of Chemistry and Biochemistry, Faculty of Sciences, University of Lisbon, 1749-016 Lisbon, Portugal; 3 Diamond Light Source, Harwell Science and Innovation Campus, Didcot, OX11 0DE UK

**Keywords:** FT-IR imaging, Fourier transform infrared, High definition, Cell systems/single cell analysis, Immersion objective, Anti-cancer drugs

## Abstract

FTIR imaging is a label-free, non-destructive method valuably exploited in the study of the biological process in living cells. However, the long wavelength/low spatial resolution and the strong absorbance of water are still key constrains in the application of IR microscopy ex vivo. In this work, a new retrofit approach based on the use of ZnS hemispheres is introduced to significantly improve the spatial resolution on live cell FTIR imaging. By means of two high refractive index domes sandwiching the sample, a lateral resolution close to 2.2 μm at 6 μm wavelength has been achieved, i.e. below the theoretical diffraction limit in air and more than twice the improvement (to ~*λ*/2.7) from our previous attempt using CaF_2_ lenses. The ZnS domes also allowed an extended spectral range to 950 cm^−1^, in contrast to the cut-off at 1050 cm^−1^ using CaF_2_. In combination with synchrotron radiation source, microFTIR provides an improved signal-to-noise ratio through the circa 12 μm thin layer of medium, thus allowing detailed distribution of lipids, protein and nucleic acid in the surround of the nucleus of single living cells. Endoplasmic reticula were clearly shown based on the lipid ν(CH) and ν(C=O) bands, while the DNA was imaged based on the ν(PO_2_^−^) band highlighting the nucleus region. This work has also included a demonstration of drug (doxorubicin) in cell measurement to highlight the potential of this approach.

**Electronic supplementary material:**

The online version of this article (10.1007/s00216-018-1245-x) contains supplementary material, which is available to authorized users.

**Figure:**
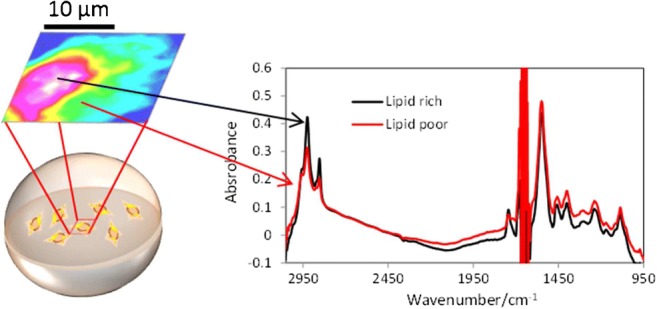
Graphical abstract

## Introduction

MicroFTIR is a chemically specific, non-destructive analytical method which does not require any external labelling for molecule tracking. IR spectroscopy advantageously uses low energy photons thereby avoiding unwanted fluorescence, photo-bleaching or light-induced sample damage. Measured IR absorbance follows the Beer-Lambert’s law and therefore FTIR can be used as a quantitative technique. Significantly, it can be used to analyse living cells in situ without interfering on natural biological process. Monitoring living cells in their culture medium by FTIR imaging has been recognised as a powerful method for living cell studies [1–10]. Information obtained from living cells is more physiologically significant than from fixed cells. Light scattering from live cell measurement is inherently far less severe compared to dried cell measurements because of the smaller difference in refractive index between the cell and the surrounding medium [11].

When measuring live cells by FTIR, three major challenges to be considered are the strong IR absorbance of water in the cell culture medium and within the cell itself, the biocompatibility of the IR substrate and the limited spatial resolution relative to visible microscopy and in terms of subcellular structures. To experimentally overcome the strong absorbance of water, a number of approaches have been derived including using the attenuated total reflection (ATR) method, which probes the attached living cell without significant contribution from the water in the culture medium [3, 4, 6, 7], the use of a small path length liquid cells or microfluidic transmission cell with spacer matching to the thickness of the cell [12–15]. Synchrotron-based FTIR microscopy is also often used to achieve diffraction limited resolution and to enhance the signal to noise ratio [5, 12, 16–21]. In ATR mode, the live cell layer attached to the ATR element absorbs the evanescent wave generated during the internal reflection, which probes up to a few micrometres into the cell depending on the refractive index, *n*, of the element and the sample, and the angle of incidence [22]. Germanium was found to be a suitable substrate for cell attachment, and its high refractive index significantly increases the numerical aperture of the optical system (up to fourfold) which allows the imaging of subcellular features [3, 22]. However, the high refractive index of Ge also results in a relatively small depth of penetration and path length therefore the spectra may neither be presentative of the cell bulk content nor have high absorbance signal. Furthermore, we recently found that bare Ge element can be damaged by the attached living cells and a coating is needed to reduce the damage [23]. When higher SNR and a full bulk measurement of cells is desirable, transmission mode measurement should be used. In this mode, living cells are seeded on an infrared transparent window and confined into a liquid cell by two windows. Infrared spectra of individual single living cells can be measured using an infrared microscope by either mapping with an aperture size matching or smaller than the size of the cell or with an imaging focal plane array detector [20, 21, 24]. However, microscopic imaging measurement through a standard IR liquid transmission cell with standard IR windows (usually 4–6 mm thick) has shown to be heavily affected by chromatic aberration due to refraction of IR light at the air-window interface [25, 26]. By placing a CaF_2_ lens on the transmission cell window with a correct centre thickness to create a “hemispherical lens” on the sample, the chromatic aberration can be eliminated with the added advantage of increasing the magnification and spatial resolution of the resultant image [27]. It has been shown that this approach allows imaging of single cells in wax printed microfluidic channels in a standard transmission IR cell [12]. The improvement on spatial resolution via the “hemisphere” was found to be determined by the refractive index of the lens, i.e. ~ 1.4 times when CaF_2_ was used. It is expected that a higher magnification and spatial resolution can be obtained when a hemisphere with higher refractive index is used. ZnSe is a popular IR transparent substrate that has a refractive index of 2.4 but was found to be not compatible to a large number of cell lines [28]. ZnS, in contrary to a recent article [29], was found to be highly compatible to a large number of cell lines [6, 7, 28] and has a high refractive index of 2.25. Using ZnS hemispheres in FTIR microscopy, therefore, should increase the spatial resolution by 2.25-fold, allowing to achieve subcellular FTIR image of live cells in transmission mode, which is not demonstrated before. While the method improves the overall quality of the image and spectrum, the SNR could be limited due to the strong absorbance of water in the mid-IR region and the small aperture required for high spatial resolution measurement. The higher refractive index of ZnS (compares to CaF_2_ and when used without anti-reflection coating) also increases reflection loss potentially resulting in a lower throughput of light. The main aim of this study is to demonstrate the combined ZnS hemispherical lens approach with the synchrotron radiation that can maximise the signal and achieve both spectral quality and spatial resolving power for live subcellular studies. As a first step, we measured the 1951 United State Airforce (USAF) resolution target to demonstrate the achieved lateral spatial resolution with two different aperture and step size. Then, we image the distribution of different chemical components in full thickness living cells to identify the subcellular organelles in the surrounding of the nucleus using synchrotron FTIR microspectroscopy as an example of the potential applications. This will pave the way to study in situ drug interactions with live cells for the monitoring of both drug and biochemical changes in cells during drug treatment at subcellular level.

## Method

### Synchrotron FTIR microscopy

The experiment was carried out at the B22 beamline (*MIRIAM*) of the Diamond Light Source synchrotron facility. The system is comprised of a Vertex 80v FTIR spectrometer and a Hyperion 3000 microscope system (Bruker optics) with a 36× reverse Cassegrain reflective objective (NA = 0.5 in air) and a matching condenser. A high sensitivity mid-band mercury cadmium telluride (MCT) single element detector was used for the mapping experiment. This is 50 μm pitch size and has a cut-off at circa 650 cm^−1^. A multilayer Ge filter was used to reduce the spectral range below 4000 cm^−1^ of the incoming beam, and the detector non-linearity was software corrected during acquisition.

### Resolution target measurement

An 8-mm diameter ZnS hemisphere was placed directly on the 1951 USAF resolution target (Newport Corporation) with the centre of the hemisphere aligned to the group 7 element 6 feature which contains 228.1 line pair per mm, i.e. line width of 2.19 μm. The signal was optimised in reflection mode on a reflective area ~ 15 μm away from the line feature where a background measurement was also measured. Water was added to fill the gap between the hemisphere and the resolution target to improve the visibility of the feature. Images were obtained by scanning using a 6 μm × 6 μm aperture size in air (i.e. 2.7 μm × 2.7 μm through the ZnS hemisphere) with a step size of 2 μm (i.e. 0.89 μm through the ZnS hemisphere) and a set aperture size of 3 μm × 3 μm in air (i.e. 1.3 μm × 1.3 μm through the ZnS hemisphere) with a set step size of 1 μm (i.e. 0.44 μm through the ZnS hemisphere). Measurements were obtained by averaging 16 scans at 4 cm^−1^ spectral resolution giving a scanning time of 2–3 s per spectrum.

### Live cell measurement

A549 cells (86012804 Sigma) were used in this experiment. Cells were grown in the Diamond B22 cell culture lab nearby the beamline. They were DMEM supplemented with 10% foetal bovine serum (FBS), 2 mM L-glutamine and 100 unit/mL penicillin streptomycin in a T25 tissue culture flask and incubated in a 5% CO_2_ incubator. Cells are harvested when reached ~ 80% confluency. The harvested cells were suspended in fresh DMEM medium at 100,000 cells per mL and 100 μL of the cell suspension was seeded directly on the flat side of the ZnS hemisphere. The ZnS hemisphere carrying the cell suspension was incubated in the CO_2_ incubator for 12–24 h to allow the cells to attach to the ZnS surface. Once the cells are attached (observed through an inverted optical microscope), the DMEM medium was removed and CO_2_-independent L15 medium, supplemented with 10% FBS, 2 mM L-glutamine and 100 unit/mL of penicillin streptomycin, was added to the attached cell. The ZnS hemisphere with the attached cells was carefully loaded into a prototype transmission device where the cells are then sandwiched between the two ZnS hemispheres with a 12-μm spacer. The prototype can hold ~ 200 μL of medium to maintain the viability of cells during measurement. Once the hemispheres were aligned in the microscope, a background was measured in an area with just medium but no cell attached. (Note that the medium contains mostly water with 0.3 wt% amino acids, 0.4 wt% protein, 0.02 wt% phosphate and 0.1 wt% carbohydrates, i.e. the compounds that may interfere with the spectrum are at a much lower concentration than in the living cell). A typical spectrum measured from the background region is shown in Electronic Supplementary Material (ESM) Fig. S1, which demonstrate the lack of interference from any organic compounds. FTIR images of the cell between the two ZnS hemispheres were then captured by mapping: i.e. sample scanning via 23 μm × 23 μm aperture size in air (i.e. 10 μm × 10 μm through the ZnS hemisphere) with a step size of 7 μm (i.e. 3.1 μm through the hemisphere) and a 6 μm × 6 μm aperture size in air (i.e. 2.7 μm × 2.7 μm through the hemisphere) with a step size of 2 μm (i.e. 0.89 μm through the hemisphere). Measurements were obtained by averaging 50 scans at 4 cm^−1^ spectral resolution giving a scanning time of 10 s per spectrum. Cell viability in the prototype transmission device for more than 12 h was confirmed using trypan blue assay (ESM Fig. S2).

## Results and discussion

### Resolution target imaging

**Fig. 1:**
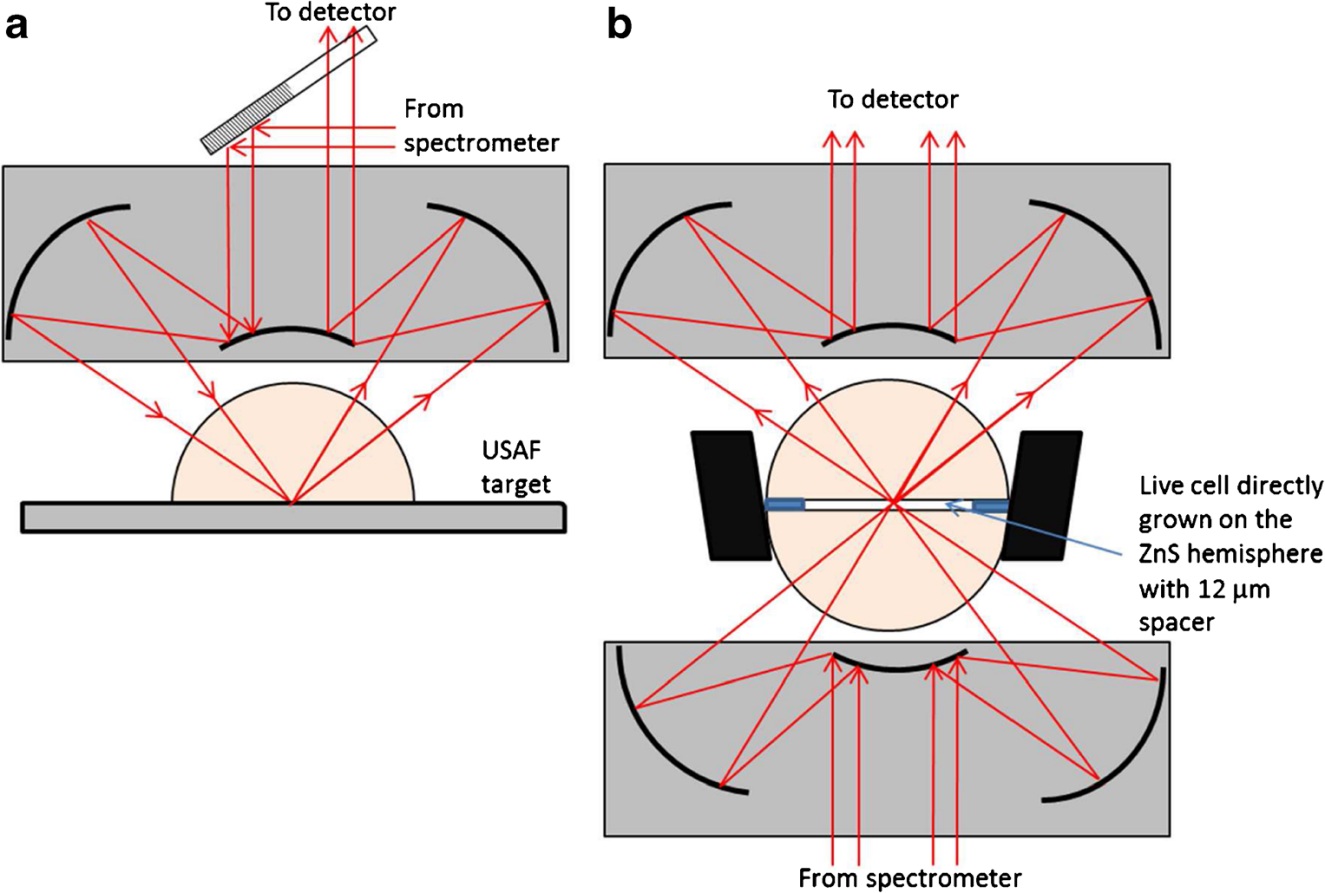
Schematics showing (**a**) the ZnS hemisphere on the USAF target where measurements were made in transflection mode and (**b**) the prototype transmission cell where live cells were sandwiched between two ZnS hemisphere with a 12-μm thick spacer and the measurements were made in transmission mode

The resolution test was carried out in reflection mode as the features on the USAF target were printed on a thick (~ 2 mm) chromium plated glass slide which prevents the use of a matching ZnS hemisphere on the condenser side underneath the sample—required in transmission mode to obtain a good throughput of light (Fig. 1a). In reflection mode, the angle of incident at the ZnS/sample interface from the 0.5 NA objective is smaller than the critical angle; therefore, the measurement is made in transflection mode, not attenuated total internal reflection mode. The throughput of light was found to be high enough for the use of small aperture, which is critical to obtain high spatial resolution images. However, in reflection mode, the same objective is used for illumination and collection of light and the microscope is designed to achieved that by illuminating half of the objective to focus light onto the same while the other half to collect the light as shown in Fig. 1a. With this design, we have previously shown that the lateral spatial resolution along vertical axis will be at least twice lower [22]. Nevertheless, the experiment enabled the estimation of the lateral spatial resolution through the horizontal axis where the objective was fully illuminated.

**Fig. 2:**
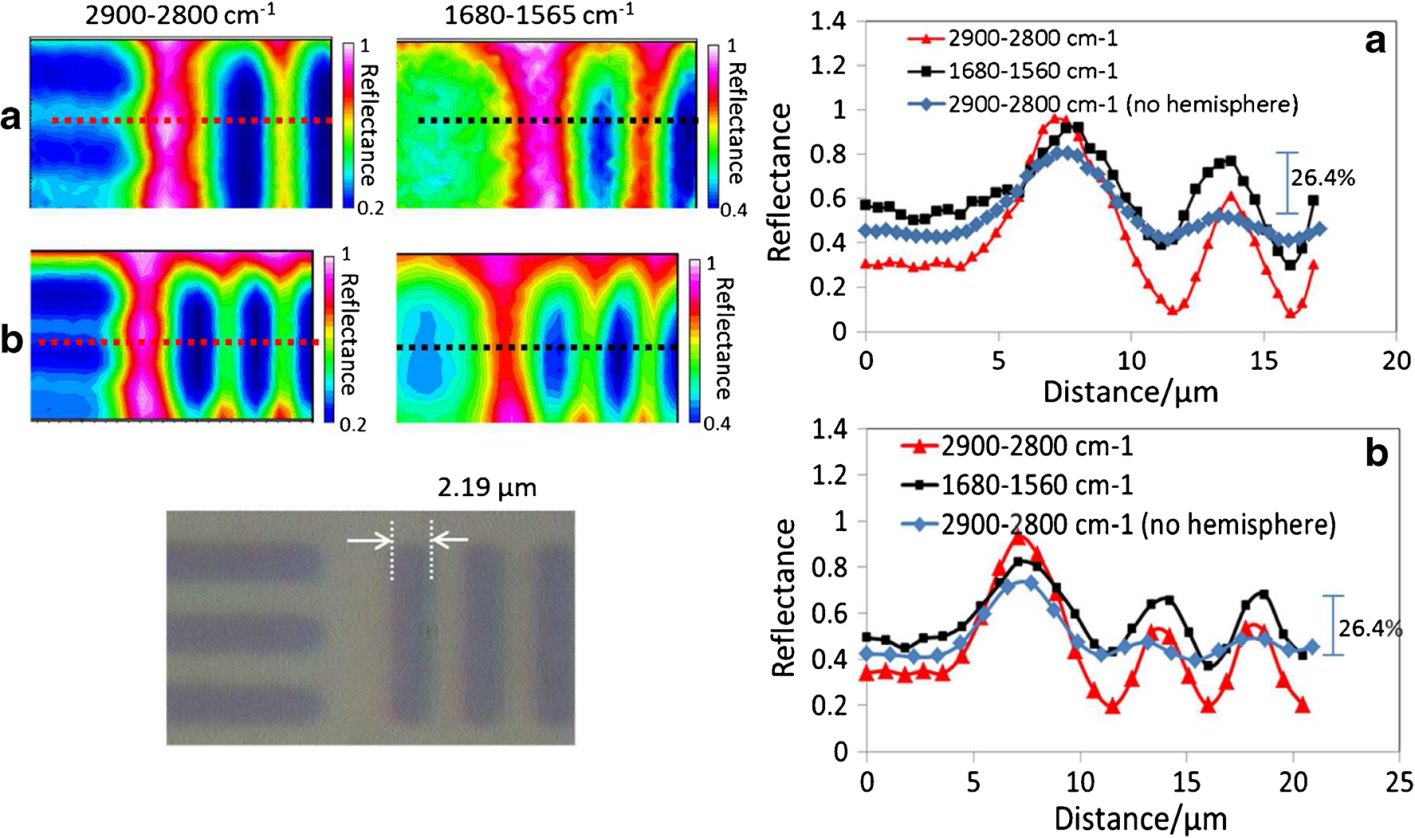
FTIR, visible images and extracted transmittance profiles of the 1951 USAF resolution target measured through the ZnS hemisphere with (**a**) effective aperture size of 1.3 μm × 1.3 μm with an effective step size of 0.44 μm and (**b**) effective aperture size of 2.7 μm × 2.7 μm with a set step size of 0.89 μm for the black and red profiles. A blue profile is also added to the plots, which represents the measurement made without the hemisphere with (**a**) 1 μm × 1 μm with an effective step size of 0.4 μm and (**b**) 3 μm × 3 μm with an effective step size of 1 μm

The Rayleigh criterion, which was originally developed to define the resolving power of an optical system, was used for comparison purpose. When imaging two adjacent points with incoherent illumination and a circular aperture, the criterion for “just resolved” is when the maximum of the airy disc of one of the point overlaps with the first minimum of the airy disc of the other point. In this situation, the intensity at the middle between the two points will be ~ 0.736 of the maximum which produce a peak to trough intensity variation of ~ 26.4%. Although the situation with the imaging of the USAF target is not the same (the target was designed to be used as a qualitative measurement of spatial resolution), the criterion was found to be a useful guide for resolution comparison purpose between various imaging systems. FTIR images of the 1951 USAF resolution target measured at two spectral regions are shown in Fig. 2. Two spectral regions were examined. The 2900–2800 cm^−1^ and 1680–1560 cm^−1^ regions are respectively relevant to the lipids and proteins or fatty acids bands, which are important in organic matter analysis. The group 7 element 6 vertical line features with a line thickness of 2.19 μm are clearly shown in all images and, as expected, the vertical line features are better resolved than the horizontal line features in all cases. The extracted profiles have shown that the vertical lines are all clearly shown, i.e. resolved. When using the 6 μm aperture with a 2-μm step size, the effective aperture size is reduced by the factor of the refractive index of the hemisphere (2.25 for ZnS) to 2.7 and 0.89 μm, respectively. With this setting, the 2.19 μm features were just resolved in at the wavelength range of 1680–1560 cm^−1^ (*λ* ~ 6 μm) giving a spatial resolution of *λ*/2.7. There is a further improvement in the sharpness in the profile with contrast of ~ 40% with the same wavelength range when the aperture and step size are halved (Fig. 2a). However, the time taken for obtaining the image of the same area with the smaller aperture setting was quadrupled while the throughput of light was reduced by more than fourfold which makes it less practical for live cell studies but could be useful for other applications where the sample is stationary. The profiles measured using the 2900–2800 cm^−1^ range without the hemisphere with comparative effective aperture and step size are also added to Fig. 2a (aperture size of 1 μm, step size of 0.4 μm) and 2B (aperture size of 3 μm, step size of 1 μm) for comparison. In both cases, the lines were not resolved without the hemisphere with contrast well below the 26.4% criterion.

Importantly, the results have demonstrated a significant improvement over previous non-ATR-based high resolution FTIR imaging studies that also published the contrast profiles of the USAF target including using multi-beam synchrotron imaging [30], CaF_2_ hemispheres [27] or the high magnification approach in transmission mode [31, 32]. A similar improvement in spatial resolution was previous observed in ATR mode [33] and more recently when a Si immersion lens was used in back scattering mode (non-USAF target measurement) [34]. However, the latter work was not demonstrated with live cells but with polystyrene beads. While ATR mode has the potential to achieve higher spatial resolution, especially with a Ge lens [31], it is important to note that ATR mode measures the surface layer of the attached cell and the layer is thin with high refractive index elements (e.g. Ge with an angle of incidence of 30° for NA of 0.5 or 37° for NA of 0.6 produces a depth of penetration of ~ 1.1 and 0.8 μm, respectively, in the cell layer even at the longer wavelength of 1000 cm^−1^). This is in contrast to the full thickness of the sample (e.g. living cell) is measured in transmission mode. Also, the objective is half illuminated in ATR mode in a similar way as the transflection mode measurement demonstrated in Fig. 2. This is in contrast to the fully illuminated objective for the measurements made in transmission mode, as in the case of live cell measurement presented later and illustrated in Fig. 1. The high lateral spatial resolution is therefore maintained, unlike in ATR or transflection mode, along both vertical and horizontal axes of the image.

The improvement in the spatial resolution is due to the increase in NA by introducing the ZnS hemisphere in the path of the IR beam just above the sample. Interestingly, the NA of the objective is 0.5, which is increased to 1.125 through the ZnS hemisphere, and the expected spatial resolution is calculated to be 3.3 μm based on the Rayleigh criterion. However, the results have clearly demonstrated that the spatial resolution exceeded the estimation using the Rayleigh criterion by at least 30%, which is in agreement with previous works by others using reverse Cassegrain objective with an FPA detector [30]. While that previous work was based on focal plane array detector, this work has also demonstrated a similar better-than-Rayleigh criterion spatial resolution can be observed by the mapping approach.

### Live cell imaging

**Fig. 3:**
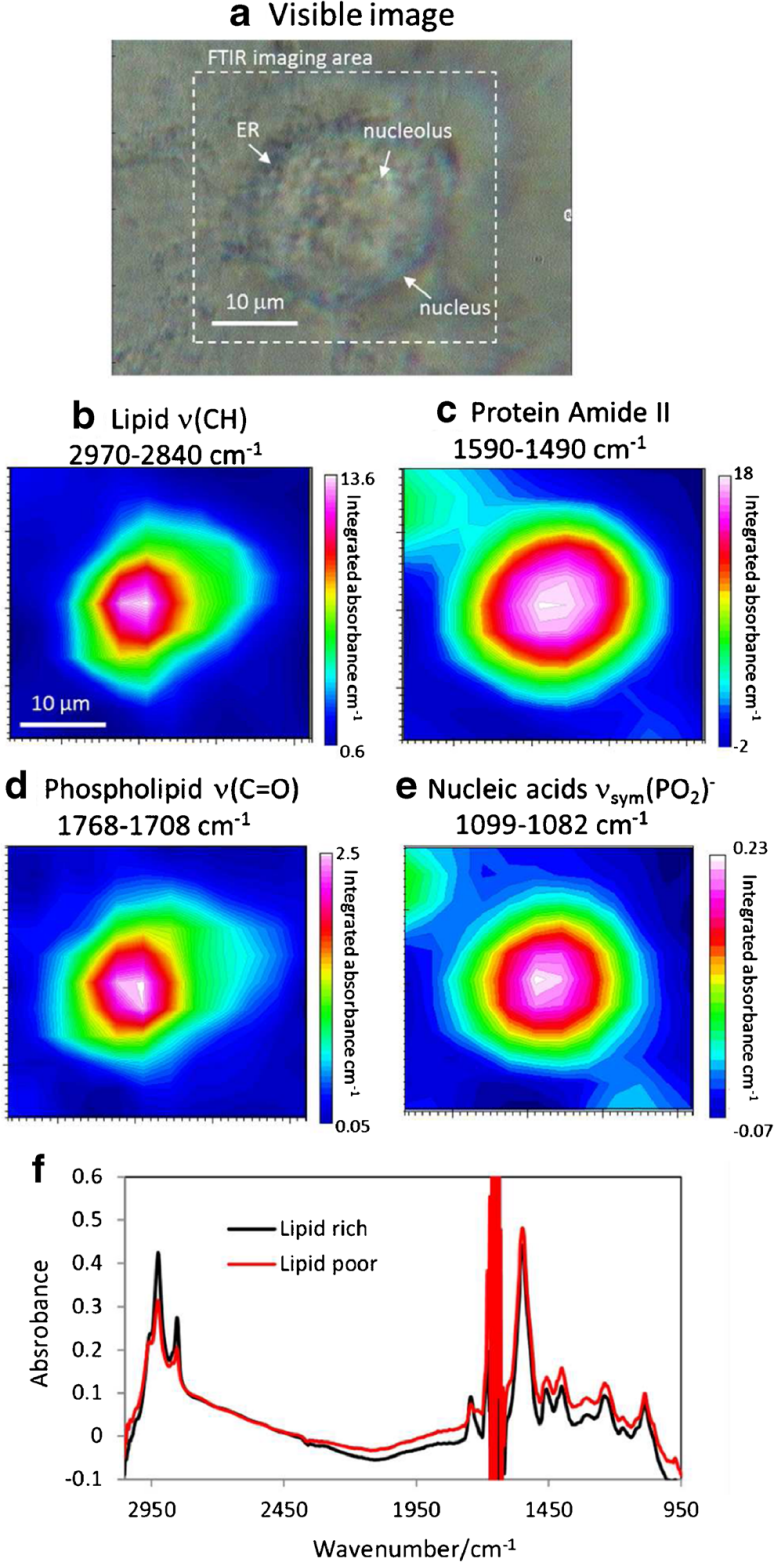
Visible imaging (**a**) and FTIR images (**b**–**e**) of the living A549 cell in the region surrounding the nucleus in the ZnS hemispheres transmission device using an effective aperture size of 10 μm. Integration range 2970–2840 cm^−1^, 1768–1708 cm^−1^, 1590–1490 cm^−1^ and 1099–1082 cm^−1^ respectively represent the distribution of overall lipid (**b**), fat/phospholipids (**d**), the overall protein (amide II) (**c**) and nucleic acids (**e**). (**f**) shows the extracted spectra from the lipid rich (black line) and lipid poor (red line) areas spectra from positions 1 and 2 shown in **b**

Figure 2 has shown that the 1.3 μm effective aperture can produce a slightly better spatial resolution than the 2.7 μm effective aperture. However, the time for mapping a large cell will be fourfold slower and therefore the 2.7 μm effective aperture was used in all live cell measurements. The speed of the imaging measurement can be increased by using a focal plane array (FPA) detector. Detailed comparisons between FPA and synchrotron system can be found elsewhere [35]. However, combining synchrotron source with an FPA has shown to produce benefit only at high magnification objectives or over small field of view areas [13] because of the diffraction limited microbeam, except when the unique multi-beam approach is used [30]. Furthermore, the benefit of the tightly focused source with the aperture on spatial resolution will be lost. Subsequently, the use of synchrotron source IR is advantageous at high magnification by exploiting image oversampling [13, 30]. In this study, a comparison is made with the live cell imaged using the current standard synchrotron mapping protocol in transmission (Fig. 1b), which uses a 10 μm effective aperture with 3 μm step size and the results are shown in Fig. 3.

The results have shown that the living cell can be easily located by plotting the amide II absorbance (1590–1490 cm^−1^) across the mapped region. In this experiment, the imaging area is focused around the nucleus region of the cell where the thickness of the cell is greatest and many large intracellular organelles are located. The image shows the amide II, a key protein band, was used here rather than amide I because the absorbance of water with this transmission cell masked the amide I spectral region. During this experiment, only a slight pressure was applied between the two hemispheres to avoid physical damage to the living cells. There are rooms to refine the experimental procedures to incrementally increase the pressure to reduce the path length through the medium above cell for a better SNR. This will be refined in the future development of the prototype transmission device. The use of a thinner spacer will also reduce the contribution from the medium above the attached cell further improve the SNR of the measurement. Previous works have shown that cell remains viable for several hours when sandwiched between windows with a gap of a few μm [20, 36] and > 60 h when subjected in a flow device [15]. Nevertheless, the current procedure enables the demonstration of the principle of subcellular imaging of living cells with the improved spatial resolution.

It is interesting to note that the distribution of the overall lipid represented by the CH_2_ and CH_3_ stretching mode vibration in the 2970–2840 cm^−1^ region has shown a smaller, more localised distribution than then the amide II-based protein map suggesting a lipid-rich organelle is present near the middle of the cell. The representative spectra (ratioed to the background spectrum measured from a near-by area that is not occupied by cells) extracted from the lipid-rich and lipid-poor region of the cell are shown in Fig. 3. While the amide II band shows a similar absorbance, the lipid bands from the lipid-rich region show a stronger absorbance than from the lipid-poor region, which demonstrates the images shown reflect the differences in the spectra. Compared to the earlier study where CaF_2_ hemispheres were used for live cell imaging, which has a spectral range cut-off at around 1100 cm^−1^, Fig. 3 shows that the spectral range can reach down to 950 cm^−1^ when using the ZnS hemispheres, which is critical for the study of DNAs (1087 cm^−1^ and ~ 970 cm^−1^) and carbohydrates such as glycogen (~ 1150–1000 cm^−1^).

**Fig. 4:**
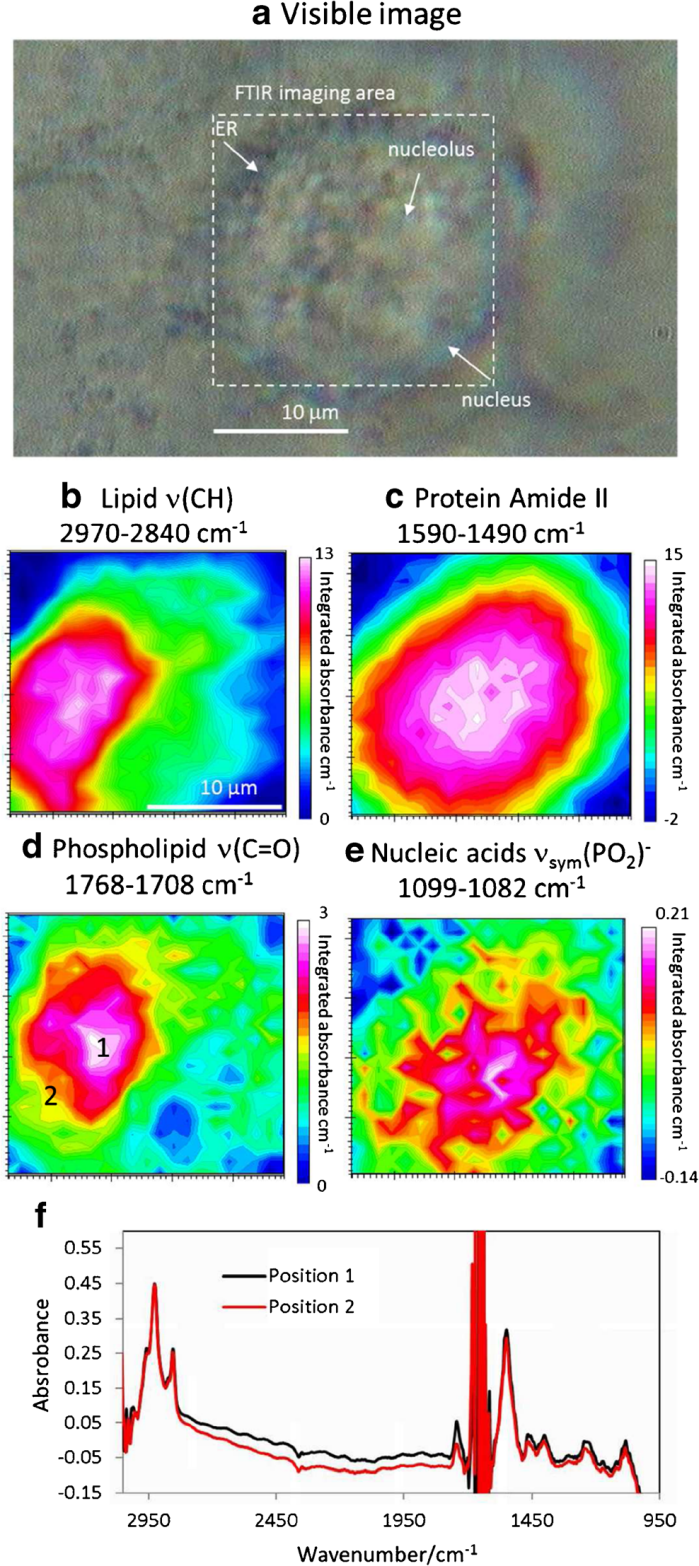
Visible imaging (**a**) and FTIR images (**b**–**e**) of the living A549 cell in the region surrounding the nucleus in the ZnS hemispheres transmission device using an effective aperture size of 2.7 μm. Integration range 2970–2840 cm^−1^, 1768–1708 cm^−1^_,_ 1590–1490 cm^−1^ and 1099–1082 cm^−1^ respectively represent the distribution of overall lipid (**b**), fat/phospholipids (**d**), the overall protein (amide II) (**c**) and nucleic acids (**e**). (**f**) shows the extracted spectra from positions 1 and 2 shown in **d**

FTIR images obtained by using the 10 μm effective aperture, however, cannot distinguish the distribution of the carbonyl containing phospholipids, which is mapped by using the 1768–1708 cm^−1^ band (peak at 1745 cm^−1^), from the general lipids (2970–2840 cm^−1^). This is expected because the size aperture used is about the same as the size of the lipid-rich organelle. The same cell was immediately mapped again using the 2.7 μm effective aperture and 0.89 μm step size, and the results are shown in Fig. 4. FTIR images generated though the amide II protein band highlighted the centre of the cell where the strongest absorbance occur at the middle. Note that the length scale of the images in Fig. 4 is smaller than in Fig. 3 because of the higher pixel density used. Comparing to the images shown in Fig. 3, the images in Fig. 4 revealed a greater detail on the lipid distribution within the cell. The image generated using the lipid ν(CH) bands in 2970–2840 cm^−1^ region has shown a clear image of the lipid-containing organelles which are mainly located to the left of the centre of the cell. Previous imaging on fixed dried fibroblast cell has also shown that detailed distribution of lipids can only be obtained when high definition approach is used, despite fibroblast cells are generally larger than the A549 cell used in this study [37]. The distribution of the carbonyl containing phospholipids (the image generated based on the band in between 1768 and 1708 cm^−1^), however, has a slightly different distribution to the overall lipids. This is also observed from the representative spectra extracted from positions 1 and 2 in Fig. 4 where both spectra show the same absorbance in the 2970–2840 cm^−1^ region but different absorbance in the carbonyl 1768–1708 cm^−1^ region. This is also shown by the extracted spectra from locations 1 and 2 on the carbonyl map. We have measured three separately cultured living cells and a similar observation was made (see ESM Fig. S3). This suggests that the lipid within the organelle is not homogeneous or there were two lipid-rich organelles next to each other. One of the largest lipid-rich intracellular organelles that is close to the nucleus is the endoplasmic reticulum (ER). It consists of membrane lipid formed tubules and cisternae which can produce strong ν(CH) and ν(C=O) bands. Some of the major functions of the ER include protein sorting and transportation by the production of vesicles (rough ER) as well as lipid synthesis (smooth ER). The FTIR images of the lipid in Fig. 4 therefore match well to the expected chemical composition, size and location of ER. The variation in the ν(C=O) bands within the lipid region might be speculated as the differences in composition of the lipid between the rough and smooth ER. Previous live cell imaging using micro ATR approach has shown that ER contains a higher amount of glycogen than the other parts of the cell, which is not observed in this case [3]. However, the previous work imaged SKOV cells which are different to A549 cells, and the sampling volume in ATR mode is different to the transmission mode used in this study. The image created from the 1087 cm^−1^ (1099–1082 cm^−1^) band, which mainly contributed from the nucleic acid of the cell, shows a localised concentration at the middle of the image. This suggests this map represents the location of the DNA cluster in the nucleus.

### Drug in live cells

**Fig. 5:**
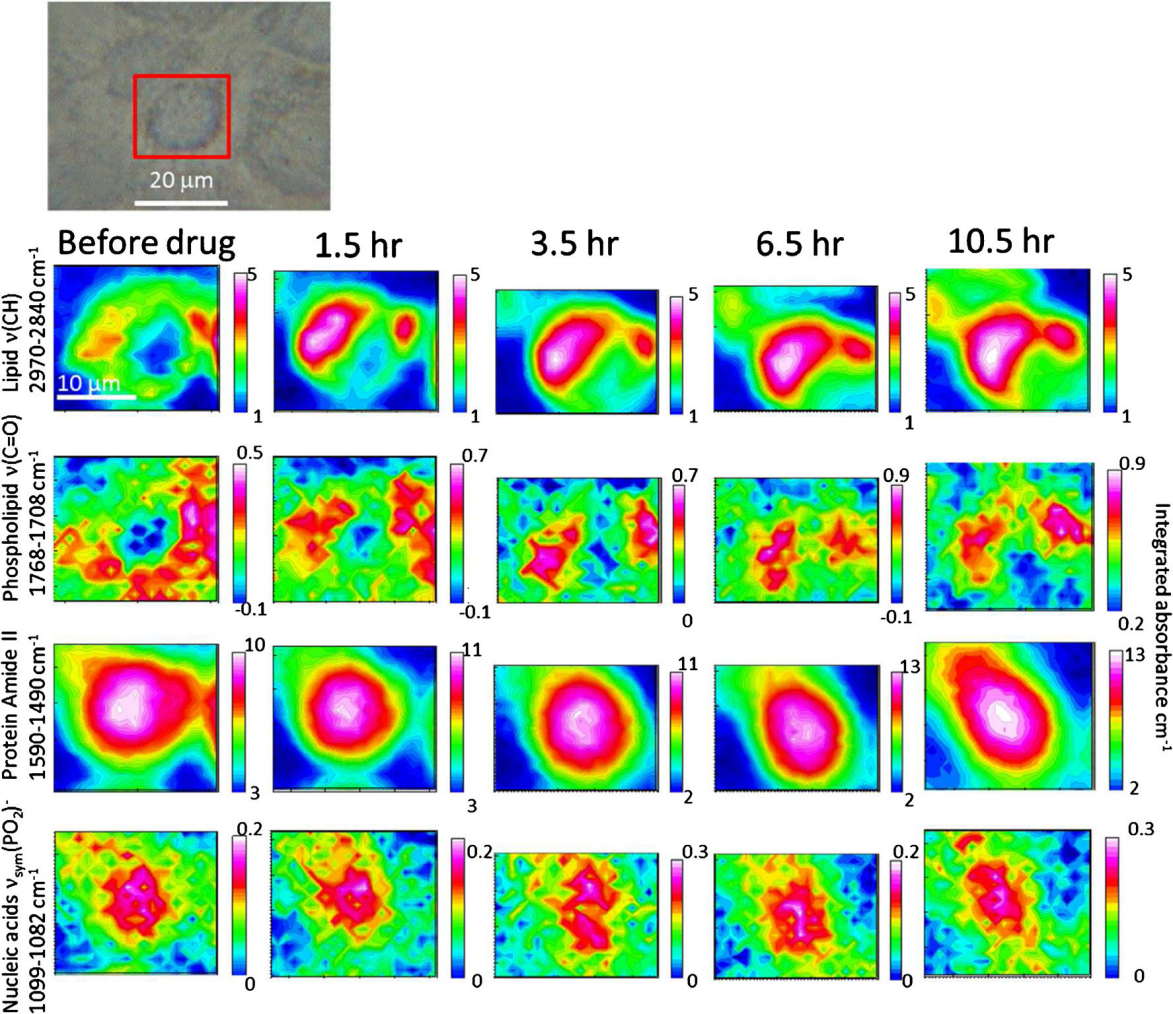
FTIR images of an A549 cell in the region surrounding the nucleus before and after exposure to 0.5 μM doxorubicin. The visible image of the measured A549 cell is shown at the top with the red square showing the approximate location where the FTIR images were measured. The colour scales used are shown on the right of each individual image

To demonstrate the potential of this approach to observe real-time subcellular changes and observe the differences between living and drug-treated cells, live cells were exposed in a well-known anti-cancer agent (doxorubicin) at IC_50_ concentration (~ 0.5 μM for 24 h exposure) [38, 39] with FTIR images of the cell captured as a function of time. The results are shown in Fig. 5. The images generated based on the ν(CH) bands show that the distribution of the lipid at the beginning was more defined in the surrounding of the nucleus of the cell, which spread towards the middle of the nucleus and to the top left part outside of nucleus of the cell after the addition of drug. The phospholipid map shows distribution similar to, but not the same as, the lipid map based on the ν(CH) bands. As mentioned previously, these regions with high ν(CH) and ν(C=O) absorbance bands, correspond to phospholipid synthesis regions of the ER. The observed spread of the lipid region can be explained by the production of vesicles which spread across the cell as a response to the drug in the early stage of apoptosis [40]. While it is not possible to determine if the cell was already dead at 10.5 h, trypan blue assay performed on the cells at the end of the experiment, which involved opening the device and injection of the trypan blue solution to the cell, has shown that most cells absorbed the blue dye, confirming cell death, in contrast to the control in ESM Fig. S2 which has shown that most cells were not stained. Similar observations were made on the repeated study based on a separately cultured A549 cell (ESM Fig. S3). The protein amide II maps and the nucleic acid band highlighted a small change in the shape of the cell, which could be a result of the natural movement of the cell. Although the trypan blue assay suggested most of the cells were dead, the causes of the relatively rapid cell death remain less clear. It was not expected all cells will die after ~ 10 h of doxorubicin treatment based on the concentration of the drug used. The relatively rapid cell death could have been accelerated by other factors such as the confined volume of the device or the disturbance to the cell during the opening of the device for the injection of the trypan blue solution. Nevertheless, this uncertainty does not neglect the results shown in this work, which demonstrated the resolution capability of the presented technique.

**Fig. 6:**
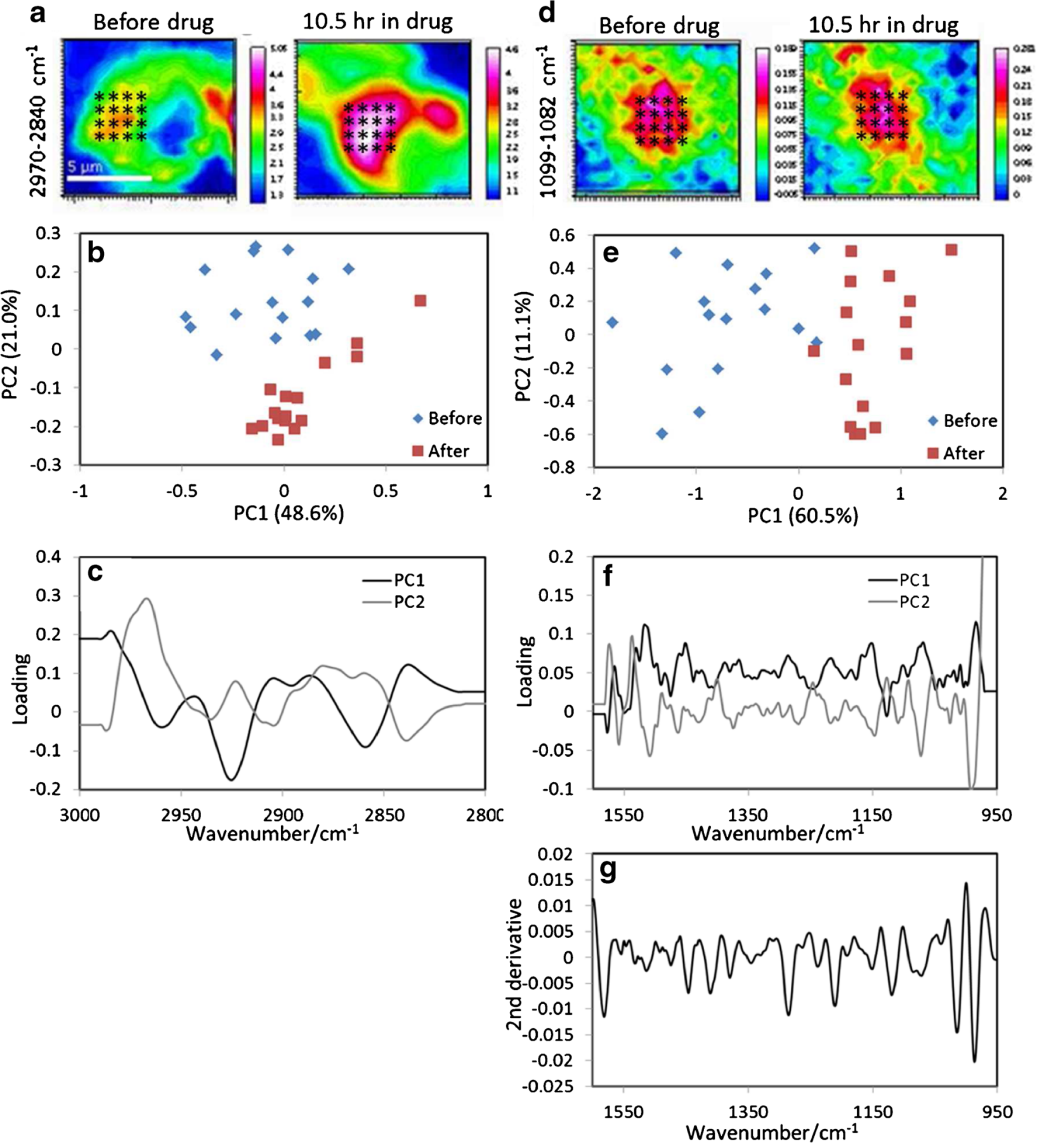
PCA analysis of the cell before and after exposing in drug for 10.5 h. The “*” marks on images in (**a**) and (**d**) show the locations of the extracted spectra. Normalised (max-min) second derivative spectra were used to calculate the PCs. (**b**) Score plots of PC1 against PC2 in the 3000–2800 cm^−1^ region from the spectra extracted from locations indicated in (**a**). Plot (**c**) is the loadings for PC1 and PC2 correspond to (**b**). (**e**) Score plots of PC1 against PC2 in the 1600–950 cm^−1^ region from the spectra extracted from locations indicated in (**d**). Plot (**f**) is the loadings for PC1 and PC2 correspond to (**d**). (**g**) A second derivative spectrum (1600–950 cm^−1^) of doxorubicin in solution form

Comparing the spectral regions where key absorbance of the cells are (2800–3000 cm^−1^ and 950–1000 cm^−1^), spectra extracted from the lipid and the nucleic acids areas (shown in Fig. 6a, d) using principal component analysis (PCA) has shown that spectra were distinguishable before and 10.5 h after the treatment of drug. Figure 6b shows that PC2 (Fig. 6c) separated the spectra extracted before and after drug treatment in the lipid-rich region. However, the bands shown in the PCs do not coincide with the lipid peak which can be interpreted as a shift in the bands between the before and after drug treatment spectra. Figure 6e shows that mainly PC1 produced the separation between the before and after drug treatment spectra in the fingerprint region. The PC1 (Fig. 6f) shows main difference can be found in the amide II and the 1000–1125 cm^−1^ regions. These changes were not observed from the raw data but were distinguished when PCA is applied. Previous studies by Byrne’s group [38, 39] on the same cell line treated in doxorubicin using Raman spectroscopy have shown that the drug accumulated in the nucleus of the treated cells. However, comparing PC1 to the spectrum of doxorubicin (2nd derivative) in Fig. 6g, there is no clear resemblance of doxorubicin spectral feature in PC1 suggesting the ability of PCA to separate the spectra before and after the addition of the drug is from the cellular responses to the drug rather than the absorbance of the drug. The lack of doxorubicin absorbance could be due to the low concentration of drug in the living cell. The changes in the 1000–1125 cm^−1^ can be a result of the action of drug, which is a DNA intercalator. It is important to note that while the results from the drug study are interesting, confirmation of the mode of action of drug, which is beyond the scope of the current work, will require more repeated experiments.

## Conclusions

Synchrotron FTIR combined with the hemispherical lens approach is a powerful method for live cell FTIR imaging with subcellular spatial resolution. The high brightness and the ability to focus the IR light to a diffraction limited spot with synchrotron has enabled the use of small apertures without significant loss in energy throughput. When combined with the use of ZnS hemispheres, which increase the NA of the objective and further reduce the diffraction limited spot-size by 2.25×, features as small as 2.19 μm were laterally resolved at ~ 6 μm wavelength, i.e. a lateral spatial resolution of at least *λ*/2.7. Subcellular features, thereby, can be imaged with the improved spatial resolution. Different images of lipids, based on the alkane chain ν(CH) band (2970–2840 cm^−1^), carbonyl band (1768–1708 cm^−1^) and DNA band (1099–1082 cm^−1^), within a living A549 cell surrounding the nucleus are were obtained. The ability to monitor the spatial distribution of various components inside the single cell in this experiment clearly demonstrated the advantages of high spatial resolution image of live cells. A preliminary test of drug treated in an anti-cancer agent doxorubicin is also shown, demonstrating the potential to study the dynamic changes in cells as a result of drug treatment. The study of changes in the same cells upon drug treatment is not possible with fixed cell measurements.

## Electronic supplementary material

ESM 1 (PDF 284 kb)
