# Supplementary material for: Subcellular mapping of living cells via synchrotron microFTIR and ZnS hemispheres
Source: Anal Bioanal Chem. 2018 Jul 21;410(25):6477–87. doi: 10.1007/s00216-018-1245-x (PMC6132686; doi:10.1007/s00216-018-1245-x)
Supplement: Supplementary file 1 — (PDF 284 kb) [file 216_2018_1245_MOESM1_ESM.pdf]

Analytical and Bioanalytical Chemistry

Electronic Supplementary Material

Subcellular mapping of living cells via synchrotron microFTIR and ZnS hemispheres

K. L. Andrew Chan, Pedro L. V. Fale, Ali Atharawi, Katia Wehbe, Gianfelice Cinque

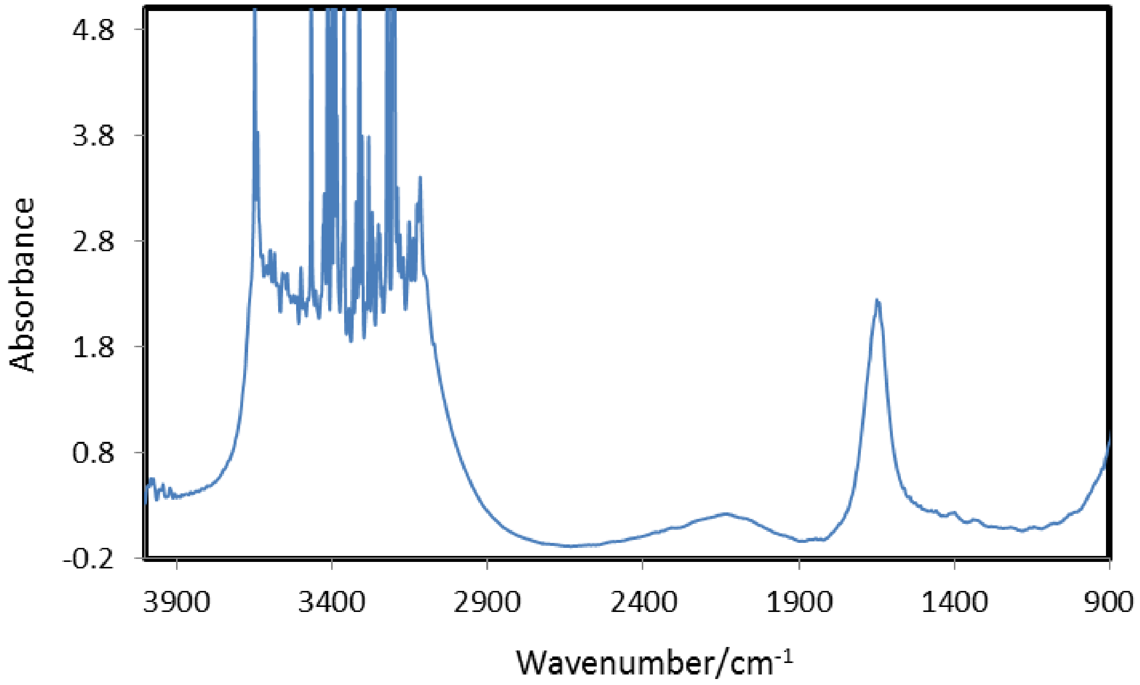

Fig. S1 An FTIR spectrum collected from a typical background area used for the live cell study. The lack of CH stretching bands and protein amide II band are indicative of the background region used is clear of cells. The periodic interference shown in the 1500-1000 cm^{-1} region is due to that the reference spectrum used to generate the spectrum shown is collected from clean hemispheres with an air gap between them, which produced the interference pattern. In the actual experiment, the background was measured with the gap between the domes filled with the aqueous medium and the interference pattern is not observed or suppressed

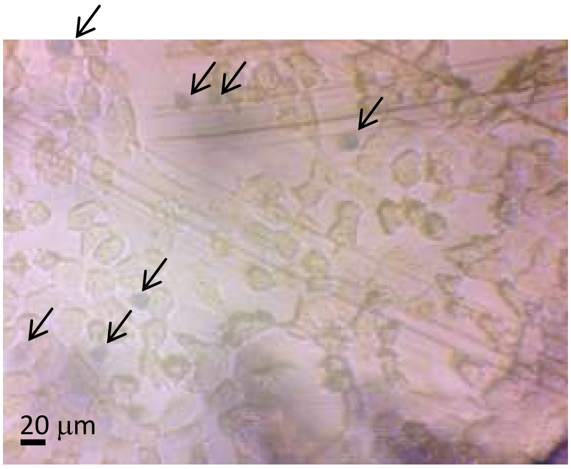

Fig. S2 A typical visible images of the A549 cells after 16 hours in the ZnS hemispheres prototype transmission device. After treatment of 0.4% trypan blue solution, ~7 blue cells (dead cell, pointed with arrows) were found within the imaged area while majority of cells (~140 cells) were still living

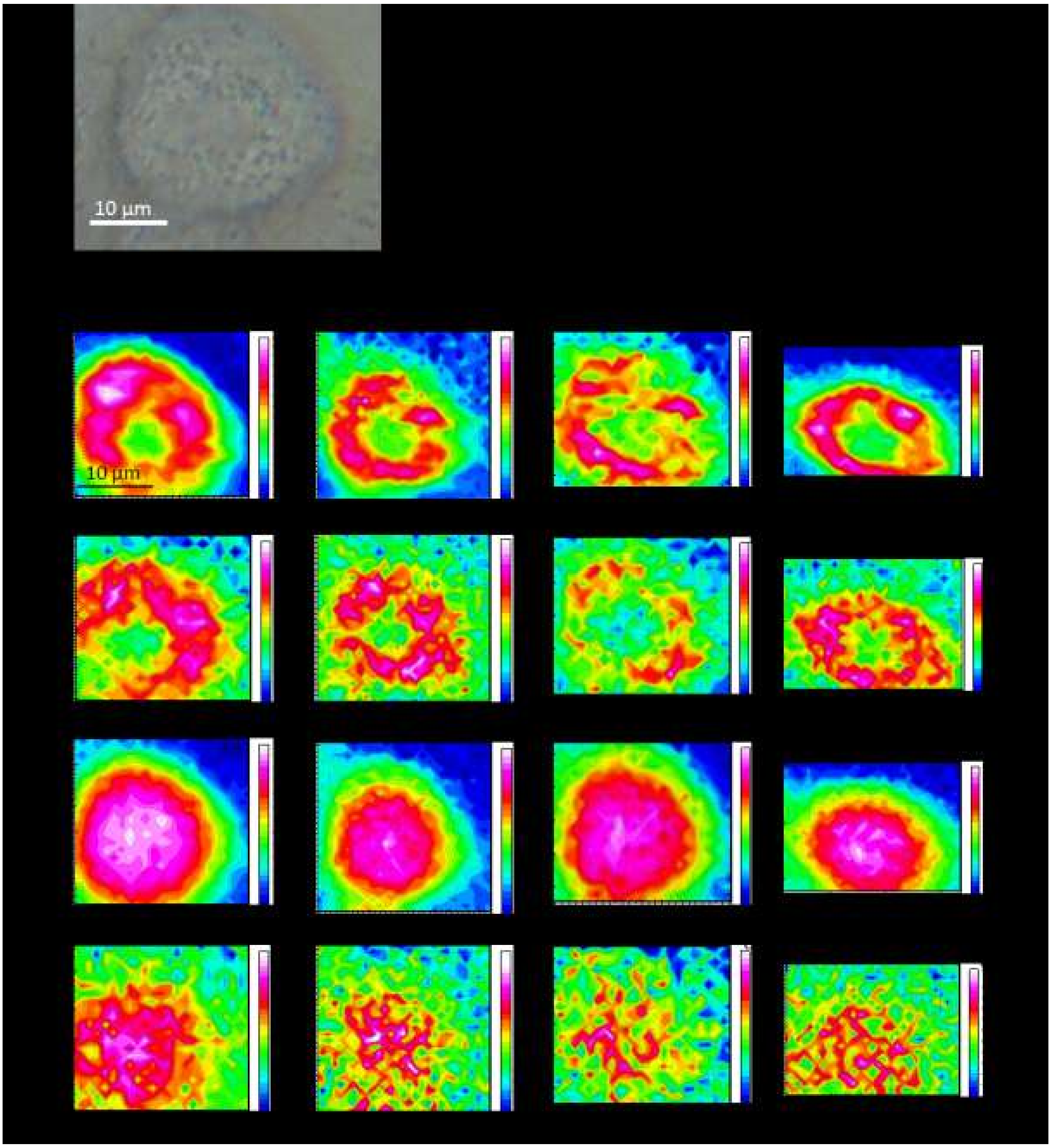

Fig. S3 FTIR images from a separate repeat experiment of A549 cell. The visible image of the measured A549 cell in the region surrounding the nucleus is shown at the top. The figure has shown a similar pattern as Fig. 5 in the main document. The colour scales used are shown on the right of each individual image
